# Knockdown of the oncogene lncRNA NEAT1 restores the availability of *miR-34c* and improves the sensitivity to cisplatin in osteosarcoma

**DOI:** 10.1042/BSR20180375

**Published:** 2018-05-28

**Authors:** Yuliang Hu, Qiuyong Yang, Long Wang, Shuo Wang, Fei Sun, Dong Xu, Jian Jiang

**Affiliations:** 1 Department of Orthopaedics, Zaozhuang hospital of Zaozhuang Mining Group, Zaozhuang, Shandong Province 277100, China; 2 Department of Orthopaedics, Affiliated Hospital of Xuzhou Medical College, Xuzhou, Jiangsu Province 221006, China

**Keywords:** Cisplatin, LncRNA NEAT1, miR-34c, Osteosarcoma, Resistance

## Abstract

Aberrant expressions of long non-coding RNAs (lncRNAs) are the culprits of carcinogenesis via regulating the tumor suppressor or oncogene. LncRNA nuclear enriched abundant transcript 1 (NEAT1) has been identified to be an oncogene to promote tumor growth and metastasis of many cancers. However, the clinical significance and function of NEAT1 in osteosarcoma (OS) remain to be discovered. We here collected OS tissues (*n*=40) and adjacent non-tumor tissues (*n*=20) to determine the expression of NEAT1 and its clinical significance. NEAT1 was overexpressed in OS tissues, which positively correlated with tumor size, Enneking stage, and distant metastasis of OS patients. The elevated level of NEAT1 was confirmed in OS cell lines including MG63 and HOS *in vitro*. Knockdown of NEAT1 by two siRNAs induced impaired cell vitalities, promoted the apoptosis, and G_0_/G_1_ arrest in two cell lines, which was associated with inhibited anti-apoptosis signals BCL-2 pathway and cell cycle-related cyclin D1 (CCND1) signals. Moreover, the tumor suppressor *miR-34c* was negatively regulated and inhibited by NEAT1 in OS. Suppression of *miR-34c* could up-regulate the expressions of its target genes *BCL-2* and *CCND1* to antagonize the effects of NEAT1 knockdown. Furthermore, overexpressed NEAT1 reduced the sensitivity of cisplatin (DDP) and inhibited DDP-induced apoptosis and cell cycle arrest via *miR-34c*. The results *in vivo* also confirmed that knockdown of NEAT1 sensitized the OS cells to DPP-induced tumor regression, delayed the tumor growth with reduced levels of Ki-67, BCL-2, and cyclin D1 signals, suggesting that NEAT1 is an oncogene and chemotherapy resistant factor in OS.

## Introduction

As the most common primary malignant bone tumor, osteosarcoma (OS) accounts for 2.4% of all malignancies in pediatric patients with a male predominance and principally comprises children and adolescents [1]. Due to the difficulty for early diagnosis of OS, the survival of patients with aggressive tumor and high incidence of metastasis to the lung still remain low (approximately 20%) [2]. In addition, the conventional therapeutic methods have reached a survival plateau due to the constitutive and acquired resistance to chemo- or radiotherapy and related life-threatening side effects [3]. Therefore, a global understanding of the underlying mechanisms of carcinogenesis would be conducive to identifying diagnostic and prognostic markers, optimizing treatment strategies and overcoming chemotherapy resistance for the patients with OS.

It is estimated that at least 90% of the genome is transcribed, with only 2% translated into proteins in humans [4]. Currently, non-coding transcripts are identified to be functional and known as non-coding RNAs (ncRNAs). Based on the their size, the heterogeneous group of molecules divided into different types of ncRNAs including miRNAs (<200 nts in length) which mediate post-transcriptional gene silencing, and long ncRNAs (lncRNAs) which are longer than 200 bp, circular RNAs (circRNAs) and Piwi-interacting RNAs (piRNAs) etc [4,5]. Accumulating evidences demonstrated that miRNAs and lncRNAs were dysregulated during carcinogenesis and function as oncogenes or tumor suppressor in many cancers [6–8], including OS [9]. The expression of tumor suppressor *miR-34c* was down-regulated in OS tumor cells to increase its target gene *RUNX2* level for cell growth of osseous cells in p53-dependent manner [10]. Overexpressed *miR-34a* and *miR-200b* in OS cell line MG-63 cells remarkably decreased Notch1 expression, resulting in the inhibition of angiogenesis, cell proliferation, and invasion of OS [11]. In hepatocellular carcinoma (HCC), lncRNA Nuclear Enriched Abundant Transcript 1 (NEAT1) functioned as competing endogenous lncRNA (ceRNA) to regulate STAT3 by sponging *miR-485* for HCC development [12]. The expressions of NEAT1 were also found to be up-regulated in prostate cancer, ovarian cancer, and breast cancer and might serve as diagnostic and prognostic biomarkers of cancer [13]. However, the clinical significance and function of NEAT1 in OS remain to be investigated.

Presently, the prognosis of cancer patients remains poor due to resistance to chemotherapy, which was involved with the dysfunction of lncRNAs and miRNAs during the treatment [14–16]. LncRNA LUCAT1 level was elevated in methotrexate-resistant OS cells. LUCAT1 knockdown suppressed the methotrexate resistance with inhibited proliferation, invasion, and tumor growth through targetting *miR-200c* and decreased the expression levels drug resistance related genes (*MDR1, MRP5, LRP1*) [17]. Besides, the expression of *miR-34c* was significantly decreased in tumor tissues from OS patients with a poor chemoresponse or metastasis. Restoration of *miR-34c* could target and decrease the Notch1 and LEF1 levels, which overcame the chemosensitivity and metastasis of OS [18].

In the present study, we focussed on the clinical significance and function of lncRNA NEAT1 in OS. The expressions of NEAT1 in OS tissues were analyzed and the roles of NEAT1 in tumor growth and chemotherapy resistance were determined by knockdown or overexpression *in vitro* and *in vivo*.

## Materials and methods

### Tumor samples

All procedures performed in studies involving human participants were in accordance with the ethical standards of the Zaozhuang Hospital of Zaozhuang Mining Group and with the 1964 Helsinki declaration and its later amendments or comparable ethical standards. The samples collection in the present study was approved by Zaozhuang Hospital of Zaozhuang Mining Group and all patients completed informed consent forms and the healthy individual recruitment were also obtained from Zaozhuang Hospital of Zaozhuang Mining Group. OS tissues (*n*=40) and adjacent non-tumor tissues (*n*=20) were collected and handled from the patients with primary OS after surgery. Due to other clinical requirements, only 20 corresponding non-tumor tissues were obtained for the analysis in the present study. The 40 pediatric patients with OSs were aged from 6 to 20 years (median: 16 years). The tumor stages were confirmed according to the Enneking staging system (ESS). According to the situation of pulmonary metastasis, OS samples were categorized into patients with (*n*=11) and without pulmonary metastasis (*n*=29).

### Cells and reagents

Four OS cell lines MG63, 143B, HOS, and Saos2 cells and one human osteoblastic cell line hFOB1.19 cells were used in the present study. The cell lines were cultured in Dulbecco’s modified Eagle’s medium (DMEM) supplemented with 10% FBS (Life Technologies, U.S.A.), which was added with ampicillin and streptomycin and cultured at 37°C, 5% CO_2_ conditions.

To knockdown or overexpress the lncRNA NEAT1, siRNA-NEAT1, pcDNA 3.1-NEAT1 or negative control were conducted by RiboBio (Guangzhou, China). The oligonucleotide sequences of *miR-34c* mimics, inhibitors, or negative control were purchased from GenePharma (Shanghai, China). For the knockdown of NEAT1 *in vivo*, lentivirus vector of siRNA-SNHG7 or negative control were conducted by GeneChem (Shanghai, China). The antibodies used in the present study including anti-Bcl-2, BAX, caspase-3, and cyclin D1, CDK2, Ki-67, and GAPDH were obtained from Cell Signaling Technology (Denver, MA) and Abcam (U.S.A.).

### Transfection

The MG63 and HOS cell lines were cultured to approximately 60% confluence in 12/96-well plates for indicated time. The transfections of siRNA-NEAT1, pcDNA 3.1-NEAT1 or *miR-34c* mimics, inhibitors or negative control were performed via Lipofectamine 2000 (Invitrogen, U.S.A.) according to the manufacturer’s instructions. After transfection for the indicated time, the cells were harvested for further experiments.

### Cell counting kit-8 assay

After the transfection as indicated, cells were harvested and washed with PBS and then cell counting kit-8 (CCK-8) (Kumamoto, Japan) mixed with DMEM was used for cell viability assay, and the absorbance was measured at 450 nm by a microplate reader.

### Flow cytometry assay

After the transfection as indicated, cell apoptosis and cell cycle were analyzed. The cells were harvested and washed by PBS. Two microliters of annexin V mixed with 2 μl propidium iodide (PI, eBioscience) were used to stain cells at 4°C for 30 min for apoptosis analysis. Alternatively, the cells were stained with PI staining solution (10 µg/ml RNase A and 50 µg/ml PI) at 4°C for 30 min in dark and the results were analyzed using a flow cytometry provided with the Cell-Quest software.

### RNA isolation and qRT-PCR

According to the standard RNA isolation protocol, total RNA from tissues or cells was extracted using TRIzol reagent (Invitrogen). Quantitative real-time RT-PCR (qRT-PCR) was performed, and the expression levels of lncRNA NEAT1 and *miR-34c* were normalized to GAPDH and U6 for gene expression, respectively.

### Preparation of cell extracts and immunoblotting

To determine the molecular expressions of apoptosis and cell cycle pathway, cells treated as indicated were collected with PBS and then lysed in RIPA buffer. Protein concentrations in fractions were determined using the BCA Protein Assay (Pierce, Rockford, IL). The proteins were separated by SDS/PAGE and transferred on to nitrocellulose membrane (Bio-Rad, Hercules, CA) which were blocked in 5% BSA-contained TBST buffer (TBS containing 0.1% Tween-20) for 1 h at room temperature, and subsequently the membrane was incubated with anti-Bcl-2, BAX, caspase-3, and cyclin D1, CDK2, and GAPDH antibodies overnight at 4°C. The membrane was washing with TBST buffer for three times, and then was incubated with HRP-conjugated secondary antibody for 1 h at room temperature. The blots were washed with TBST buffer and visualized using the ECL-Plus reagent (Millipore, Billerica, MA, U.S.A.).

### Immunohistochemistry

The Ki-67 expression in tumor tissues was analyzed via immunohistochemistry (IHC) on 2-μm-thick, formalin-fixed and paraffin-embedded specimen sections. The detailed procedure was performed according to previous study described [19,20].

### Tumor model

The xenograft model of human MG63 cells were established. MG63 cells were transfected with lentivirus vector of siRNA-NEAT1 or negative control, 2 × 10^6^ conditional MG63 cells were subcutaneously injected in rear flank of nude mice (six per group), DDP was treated i.p. twice a week for 3 weeks. The tumor sizes were measured 3 days apart and the tumor volumes were calculated: V (cm^3^) = width^2^ (cm^2^) × length (cm)/2.

### Statistical analysis

The results are analyzed by using SPSS statistical software and GraphPad Prism 5.0 software. It was considered to be significant difference when *P*< 0.05. Unpaired *t*tests or Mann–Whitney U tests were used to compare the two groups, and multiple group comparisons were analyzed with one-way ANOVA. All experiments were performed at least three times.

## Results

### Overexpressed lncRNA-NEAT1 predicts poor clinical outcome of patients with OS

**Figure 1:**
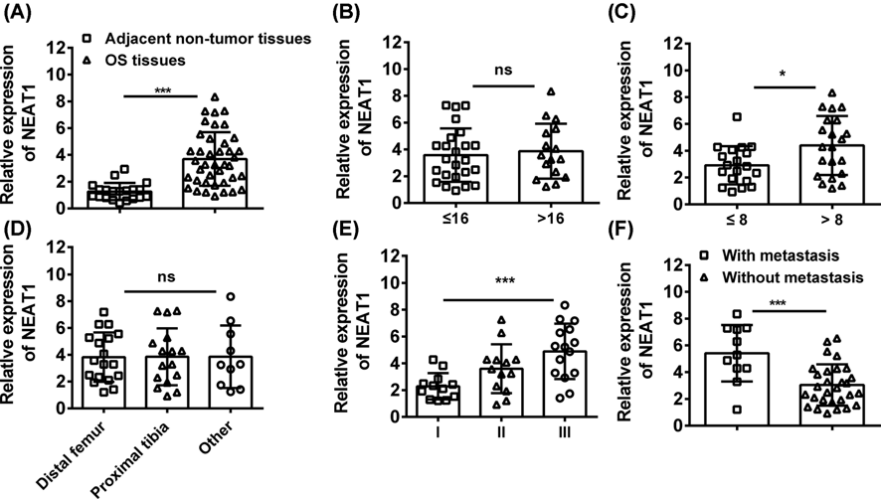
NEAT1 is up-regulated with the development of OS (**A**) The expressions of NEAT1 were determined in OS tissues (*n*=40) and adjacent non-tumor tissues (*n*=20) by Q-PCR. (**B**–**F**) The correlation between NEAT1 expression and age, tumor sites, tumor sizes, Enneking staging and metastasis were analyzed. *P<0.05, **P<0.01, data represent the means ± S.D.

To investigate the role of NEAT1 in OS in clinical, OS tissues from surgery (*n*=40) and adjacent non-tumor tissues (*n*=20) were collected. The expressions of NEAT1 in tissues were analyzed by Q-PCR and the results indicated that the OS tissues had higher level of NEAT1 than relative normal tissues (Figure 1A). Moreover, the correlations between the clinicopathological characteristics of OS patients and NEAT1 expression were determined. We found that the expression of NEAT1 was comparable in patients with different age (age range: 6–20 years, median: 16 years) (Figure 1B) and localization of the primary tumor (femur, tibia etc.) (Figure 1D). However, the patients with high Enneking staging (Figure 1E), large tumor size (Figure 1C), and distant metastasis (Figure 1F) tended to have higher expression of NEAT1 in tumor tissues. These data indicated that highly expressed NEAT1 was an oncogene and predicted a poor clinical outcome for patients with OS.

### LncRNA-NEAT1 is required for the tumor growth and metastasis of OS *in vitro*

**Figure 2:**
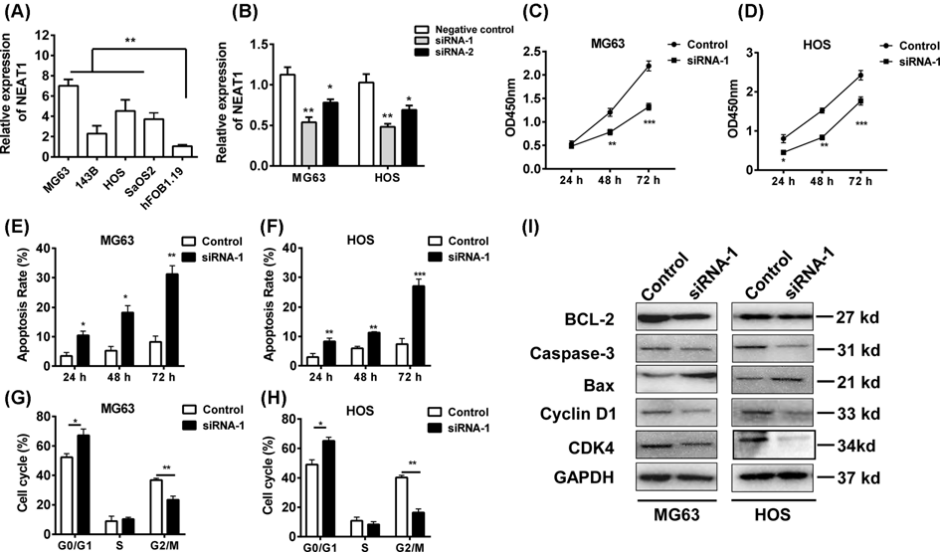
NEAT1 promotes cell growth of OS cells *in vitro* (**A**) The expression of NEAT1 in OS cell lines MG63, 143B, HOS, and Saos2 cells and one human osteoblastic cell line hFOB1.19 cells were determined by Q-PCR. (**B**) The NEAT1 was knocked down in MG63 and HOS cell lines and (**C**,**D**) the cell vitalities (**E**,**F**) apoptosis and (**G**,**H**) cell cycle of two cell lines were analyzed by CCK-8 and flow cytometry. (**I**) The expressions of BCL-2, caspase-3, BAX, cyclin D1, and CDK were analyzed by western blot in MG63 and HOS cell lines. *P<0.05, **P<0.01, ***P<0.001, data represent the means ± S.D.

Considering the clinical significance of NEAT1 in OS, we next investigated the function of NEAT1 in OS *in vitro*. Four OS cell lines MG63, 143B, HOS, and Saos2 cells and one human osteoblastic cell line hFOB1.19 cells were used and we confirmed that the NEAT1 level was up-regulated in tumor cells when compared with that in normal cells (Figure 2A). MG63 and HOS cell lines were used in further study. After the efficient knockdown of NEAT1 in two cells (Figure 2B), we selected one siRNA and the cell vitality, apoptosis, and cell cycle were assessed. The results demonstrated that inhibition of NEAT1 was capable of repressing the cell vitality in time-dependent manner (Figure 2C,D). Knockdown of NEAT1 also induced the apoptosis (Figure 2E,F) and G_0_/G_1_ arrest in MG63 and HOS cell lines (Figure 2G,H).

To identify the key regulator of these effects, we analyzed the apoptosis pathway and cell cycle signals (Figure 2I). We found that knockdown of NEAT1 inhibited the expression of anti-apoptosis factor BCL-2, reduced the caspase-3 level but up-regulated the pro-apoptosis factor BAX level. In addition, the expressions of cyclin D1 and CDK4 were also decreased by the inhibition of NEAT1. Thus, NEAT1 could regulate the BCL-2-related apoptosis pathway and cyclin D1-related pathway to promote tumor growth of OS cells.

### LncRNA-NEAT1 could inhibit tumor suppressor *miR-34c*

**Figure 3:**
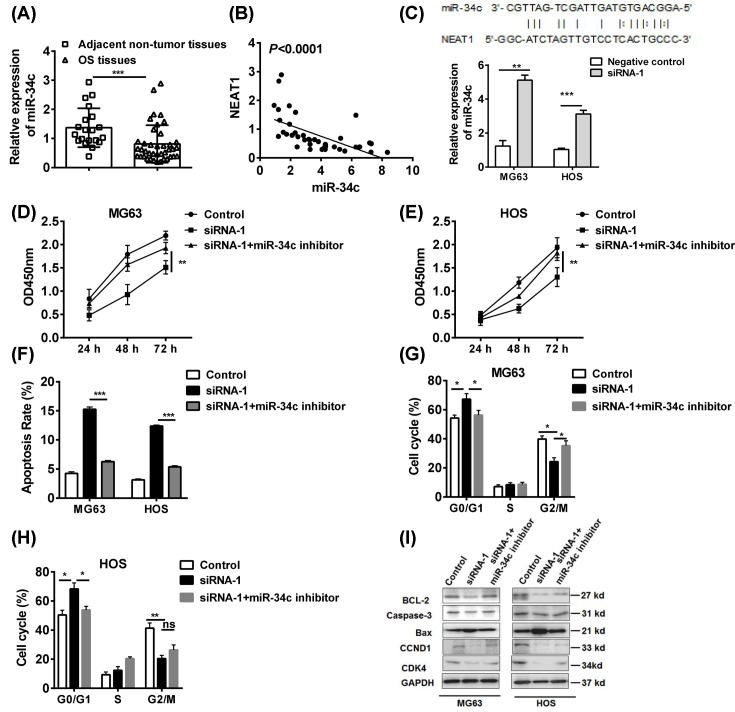
NEAT1 could inhibit tumor suppressor *miR-34c* (**A**) The expressions of *miR-34c* in OS tissues were analyzed by Q-PCR and (**B**) its correlation with NEAT1 was determined. (**C**) The expression of *miR-34c* in NEAT1 knockdown cells was assessed. After the transfection of siRNA-NEAT1 with/without miR-34c inhibitor, (**D**,**E**) the cell vitalities, (**F**) apoptosis and (**G**,**H**) cell cycle were determined by CCK-8 and flow cytometry in MG63 and HOS cell lines. (**I**) The expressions of BCL-2, caspase-3, BAX, cyclin D1, and CDK were analyzed by western blot in MG63 and HOS cell lines. *P<0.05, **P<0.01, ***P<0.001, data represent the means ± S.D.

LncRNAs are reported to exert its function by sponging the miRNAs and regulate the targets of miRNAs [21]. *MiR-34c* is a tumor suppressor in many cancers, including OS [22]. We found that the expression of *miR-34c* was down-regulated in OS tissues and negatively correlated with the expression of NEAT1 in tumor tissues (Figure 3A,B). Importantly, *miR-34c* was the predicted miRNA that directly targets the NEAT1 that was screened (http://starbase.sysu.edu.cn/index.php); and knockdown of NEAT1 in two cell lines could elevate the tumor suppressor *miR-34c* levels (Figure 3C). Although the NEAT1 inhibition impaired the cell vitality, the simultaneous inhibition of *miR-34c* could antagonize this effect and restore the cell vitality of tumor cells (Figure 3D,E). Similarly, the NEAT1 inhibition-induced apoptosis (Figure 3F) and cell cycle arrest was abrogated by *miR-34c* inhibitors (Figure 3G,H).

Interestingly, the BCL-2-related apoptosis pathway and cyclin D1-related pathway were reported to be the targets of *miR-34c* in cancer [23,24]. The results also confirmed that the *miR-34c* inhibitor restored the BCL-2 and cyclin D1 levels in MG63 and HOS cell lines (Figure 3I), which implicated that NEAT1 inhibited the tumor suppressor *miR-34c* and up-regulated cell survival signals for the development of OS.

### Overexpression of lncRNA-NEAT1 impairs the sensitivity of cisplatin via *miR-34c* in OS

**Figure 4:**
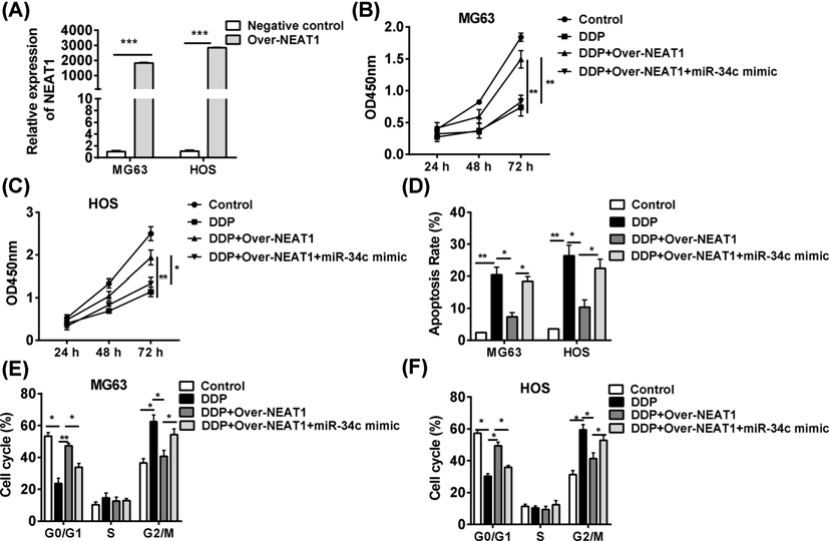
Overexpression of NEAT1 impairs the sensitivity of cisplatin via *miR-34c* in OS (**A**) NEAT1 was overexpressed in in MG63 and HOS cell lines. The cells were transfected pcDNA 3.1-NEAT1 or *miR-34c* mimics and their negative controls, and then were treated with DDP (2 μg/ml). (**B**,**C**) the cell vitalities, (**D**) apoptosis, and (**E**,**F**) the cell cycle were analyzed. *P<0.05, **P<0.01, ***P<0.001, data represent the means ± S.D.

The chemotherapy resistance is the critical cause for cancer-related deaths in OS, thus we investigated the impacts of the oncogene NEAT1 on cisplatin (DDP)-based chemotherapy. The NEAT1 was overexpressed in MG63 and HOS cell lines (Figure 4A). The results showed that overexpressed NEAT1 reduced the cytotoxicity of DDP and rescued the cell vitality, but the addition of *miR-34c* could abrogated the NEAT1-dependent DDP resistance, leading to the decreased cell vitality of two cell lines (Figure 4B,C). Similarly, overexpression of NEAT1 also inhibited DDP-induced apoptosis (Figure 4D) and G_2_/M arrest, and the *miR-34c* could abrogate the DDP resistance (Figure 4E,F). These data indicated that knockdown of NEAT1 improved the sensitivity of OS cells to DDP via up-regulation of tumor suppressor *miR-34c*.

### Knockdown of lncRNA-NEAT1 improves cisplatin-induced tumor regression of OS *in vivo*

**Figure 5:**
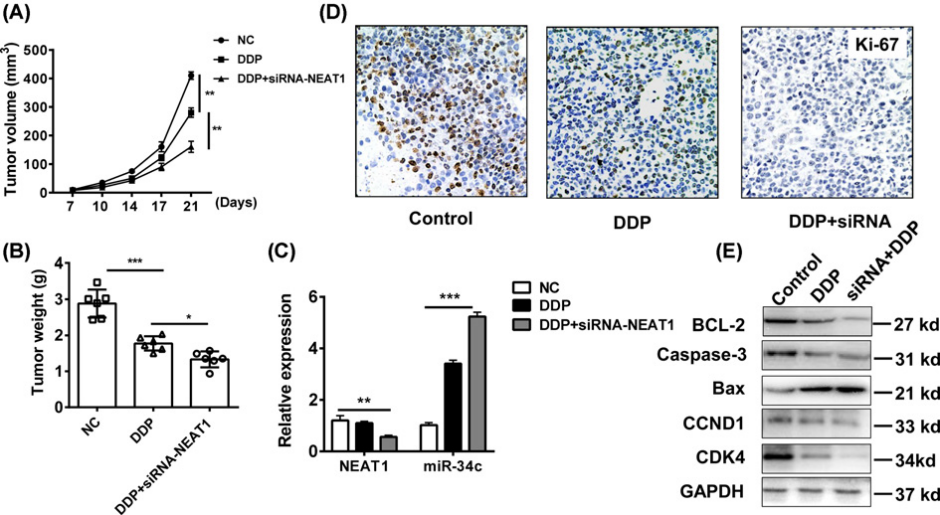
Knockdown of NEAT1 improves cisplatin-induced tumor regression *in vivo* The 2 × 10^6^ conditional MG63 cells were transfected with lentivirus vector of siRNA-NEAT1 or negative control and were subcutaneously injected in rear flank of nude mice (six per group). (**A**,**B**) The mean tumor size (mm^3^) and weight was analyzed. (**C**) The expressions of NEAT1 and *miR-34c* in tumor tissues were determined by Q-PCR. (**D**,**E**) The expressions of Ki-67, BCL-2, caspase-3, BAX, cyclin D1, and CDK were analyzed by IHC and western blot. *P<0.05, **P<0.01, ***P<0.001, data represent the means ± S.D.

To provide the evidence of oncogene NEAT1 *in vivo*, the xenograft model of conditional human MG63 cells were established. The nude mice MG63 cells were treated with/without NEAT knockdown, followed by the administration of DDP. The results showed that the mice treated with DDP and NEAT1-knockdown MG63 cells had the slowest tumor growth than that treated with DDP alone (Figure 5A). The tumor weight in the group treated DDP and NEAT1-knockdown MG63 cells also lowest than the control group (Figure 5B). Meanwhile, the expression of NEAT1 and *miR-34c* in tumor tissues were confirmed that knockdown of NEAT1 *in vivo* could up-regulate the *miR-34c* levels (Figure 5C). We further analyzed the expression of proliferation index Ki-67 in tumor tissues. NEAT1 inhibition elevated the DDP-induced inhibition of cell proliferation and suppressed the expression of Ki-67 (Figure 5D). The apoptosis and cell cycle pathway in tumor tissues were determined (Figure 5E). We found that NEAT1 inhibition plus DDP *in vivo* inhibited the BCL-2/caspase-3 levels and increased the BAX expression for tumor apoptosis, and also repressed the cyclin D1/CDK4 expression for cell cycle arrest. These findings indicated that knockdown of NEAT1 *in vivo* could inhibit the tumor suppressor *miR-34c* and sensitize the OS cells to DDP-induced tumor regression.

## Discussion

OS usually arises from the metaphysis regions of distal femur, proximal tibia, and proximal humerus. The conventional therapeutic treatments for OS relay on the surgical resection of the tumor bulk combined with chemotherapy and/or radiotherapy, which significantly improve the 5-year survival rate of OS patients to approximately 60–70%. However, the frequency of recurrence and chemotherapy resistance is high, which is the leading contributor to the decreased survival time of patients [25,26]. We here reported an oncogene lncRNA NEAT1 (NEAT1) during the development of OS in clinical. Overexpressed NEAT1 inhibited the tumor suppressor *miR-34c*, promoted the survival of OS cells, inhibited apoptosis, and reduced the sensitivity to DDP *in vitro* and *in vivo*.

Nuclear paraspeckle assembly transcript 1 (NEAT1) is a novel lncRNA localized specifically to nuclear paraspeckles, which are irregularly shaped compartments found in the nucleus’ interchromatin space. NEAT1 have been demonstrated to be up-regulated in various human malignancies and functions as oncogene in most solid tumor via sponging of tumor-suppressive miRNAs [13]. Li et al. [27] reported that NEAT1 expression in colorectal cancer (CRC) was increased in 72% cases compared with corresponding normal tissues, high expression of NEAT1 predicted poor tumor differentiation, high metastasis and TNM stage, and was an independent prognostic marker for the poor outcome of CRC patients. The expression of NEAT1 in clear cell renal cell carcinoma (ccRCC) was also found to be enhanced in tumor tissues, which positively correlated with tumor size, lymph node metastasis, and also predicted short 5-year survival rate of patients with ccRCC [28]. In this study, we also identified the oncogene role of NEAT1 in OS, and highly expressed NEAT1 in tumor tissues was associated with high tumor stage and distant metastasis. However, in acute promyelocytic leukemia, NEAT1 expression is reduced and functions as a tumor suppressor by promoting leukocyte differentiation. Thus, apart from the leukemia, NEAT1 mainly functions as an oncogene in solid tumor, including OS [13].

The miRNA sponge was the main mechanism of the function of NEAT1 during carcinogenesis. NEAT1 was found to the cell proliferation of non-small-cell lung cancer (NSCLC) though binding hsa-*miR-377* and increased the expression of its target E2F3 [29]. Tumor suppressor *miR-124* could be interacted with NEAT1 and down-regulated in nasopharyngeal carcinoma (NPC) cells, elevated NEAT1 level in tumor tissues promoted the cell growth and the progression of NPC through regulating *miR-124*/NF-κB signaling pathway [30]. In this study, we found that the tumor suppressor *miR-34c* was inhibited by NEAT1 in OS, and restoration of *miR-34c* could abrogate NEAT-1-induced proliferation and inhibition of apoptosis via regulation of the balance between BCL-2 and BAX. The role of NEAT1 in cancer chemotherapy was identified. For example, BAP1 conversely regulated the expression of NEAT-1, which contributed to sensitivity to gemcitabine in cholangiocarcinoma [31]. We here found that enhanced expression of NEAT1 impaired the sensitivity to cisplatin in OS cells, and knockdown of NEAT1 could up-regulated *miR-34c* to overcome the cisplatin resistance *in vivo*. Similarly, in the paclitaxel (PTX) resistance of ovarian cancer cells, the NEAT1 level was positively associated with the PTX resistance. NEAT1 knockdown sensitized the PTX-resistant cells to PTX via promoting PTX-induced apoptosis via *miR-194*/ZEB1 axis [32].

In conclusion, we here reported an oncogene, lncRNA NEAT1, which could predict poor clinical outcome of OS patients. Knockdown of NEAT1 up-regulated the tumor suppressor *miR-34c* to inhibit cell proliferation, induce apoptosis, and cell cycle arrest via BCL-2 and cyclin D1 pathway, which elevated the sensitivity to DDP-induced chemotherapy for tumor regression.

## Abbreviations

ccRCC: clear cell renal cell carcinoma
CRC: colorectal cancer
DMEM: Dulbecco’s modified Eagle’s medium
HCC: hepatocellular carcinoma
lncRNA: long non-coding RNA
ncRNA: non-coding RNA
NEAT1: nuclear enriched abundant transcript 1
NPC: nasopharyngeal carcinoma
OS: osteosarcoma
PI: propidium iodide
PTX: paclitaxel
TBST: TBS containing 0.1% Tween-20
